# Efficacy of two PBO long lasting insecticidal nets against natural populations of *Anopheles gambiae s*.*l*. in experimental huts, Kolokopé, Togo

**DOI:** 10.1371/journal.pone.0192492

**Published:** 2018-07-11

**Authors:** Guillaume K. Ketoh, Koffi M. Ahadji-Dabla, Joseph Chabi, Adjovi D. Amoudji, Georges Y. Apetogbo, Fantchè Awokou, Isabelle A. Glitho

**Affiliations:** 1 Insect Pest and Insect Vector Management/Ecotoxicology, Unité de Recherche en Ecotoxicologie (URET), Laboratoire d’Entomologie Appliquée (LEA), Faculté des Sciences, Université de Lomé, Lomé, Togo; 2 Noguchi Memorial Institute for Medical Research (NMIMR), University of Ghana, Legon, Accra, Ghana; 3 Programme National de Lutte contre le Paludisme (PNLP), Ministère de la Santé, Lomé, Togo; National Institute for Communicable Diseases, SOUTH AFRICA

## Abstract

LLINs containing an insecticide plus the synergist, piperonyl butoxide (PBO) have been designed for increased efficacy against pyrethroid-resistant malaria vectors. In this study, two LLINs with PBO, PermaNet^®^ 3.0 and Olyset^®^ Plus, and a pyrethroid-only LLIN, Yorkool^®^, were evaluated in experimental huts against a free-flying, wild population of *Anopheles gambiae s*.*l*. in Kolokopé, a cotton cultivated area of Togo. WHO susceptibility tube tests and subsequent molecular assays determine the *An*. *gambiae s*.*l*. populations to be resistant to pyrethroids and DDT with both target site *kdr* and metabolic resistance mechanisms involved in the resistance observed. *Anopheles gambiae s*.*s*. and *An*. *coluzzi* were present in sympatry though the *kdr* (L1014F) mutation was observed at a higher frequency in *An*. *gambiae s*.*s*. The experimental hut results showed that both PermaNet^®^ 3.0 and Olyset^®^ Plus nets induced similar levels of deterrence, exophily, and reduced blood feeding rate against wild *An*. *gambiae s*.*l*. in contrast to the pyrethroid only LLIN, Yorkool^®^. The proportion of wild *An*. *gambiae s*.*l*. killed by unwashed PermaNet^®^ 3.0 was significantly higher than unwashed Olyset^®^ Plus (corrected mortality 80.5% compared to 66.6%). Similar blood feeding inhibition rates were observed for unwashed PermaNet^®^ 3.0 and Olyset^®^ Plus; however, PermaNet^®^ 3.0 washed 20 times demonstrated significantly higher blood feeding inhibition rate than Olyset^®^ Plus washed 20 times (91.1% compared with 85.6% respectively). Yorkool^®^ performed the worst for all the parameters evaluated. In an area of pyrethroid resistance of *An*. *gambiae s*.*l* involving *kdr* target site and metabolic resistance mechanisms, LLINs with PBO can provide additional protection in terms of reduction in blood feeding and increase in mosquito mortality compared to a pyrethroid-only net, and should be considered in malaria vector control strategies.

## Introduction

Long lasting insecticidal nets (LLINs) continue to be one of the primary interventions against malaria vectors. Currently, a LLIN is expected to retain its biological activity for at least 20 standard washes under laboratory conditions and three years of recommended use under field conditions, as defined in WHO guidelines [1]. WHO Pesticide Evaluation Scheme (WHOPES) Phase II experimental hut studies are conducted on nets that pass Phase I laboratory investigations to determine comparative efficacy against free-flying, wild mosquito populations.

In Togo, insecticide susceptibility status of *Anopheles* populations was effectively reported for the first time in 2005 [2]. Pyrethroid resistance with the presence of *kdr* (L1014F) mutation was recently detected in malaria vectors in the south of the country [3]. It was also known that carboxylesterases (COEs), glutathione-S-transferases (GSTs) and cytochrome P450-dependent monoxygenases (P450s) are the three main groups of enzymes involved in the metabolic resistance to pyrethroids used for malaria vector control [4].

LLINs containing an insecticide plus the synergist piperonyl butoxide (PBO) have been designed for increased efficacy against pyrethroid-resistant malaria vectors. PBO is an inhibitor of mixed function oxidases (MFO) implicated in pyrethroid resistance, and also increases the rate of insecticide uptake through the mosquito cuticle [5]. In 2014, at the time of the evaluation, two LLINs with PBO were available: PermaNet^®^ 3.0[6] and Olyset^®^ Plus [7] PermaNet^®^ 3.0 is a LLIN with PBO and deltamethrin incorporated on polyester side panels and a mixture of deltamethrin and PBO incorporated in the polyethylene top panel. Olyset^®^ Plus LLIN is made of polyethylene netting incorporating permethrin and PBO. A third LLIN with PBO, Veeralin^®^ LN received WHOPES interim recommendation in 2016 [8]. In contrast to LLINs with PBO, the pyrethroid-only LLIN included in this study, Yorkool^®^ is a multifilament polyester net coated with deltamethrin.

In this experimental hut trials, the primary objective was to determine the comparative efficacy between LLINs with PBO and a pyrethroid-only net in an area of pyrethroid resistance with the involvement of metabolic resistance mechanisms. As per standard outcomes measures for experimental hut trials, efficacy was measured in terms of blood-feeding inhibition, deterrence, induced exophily and mortality. Characterisation of the wild mosquito population including species composition, susceptibility to the pyrethroid active ingredient in the LLINs (deltamethrin and permethrin), frequency of the target site mutation (*kdr* L1014F) and upregulated metabolic enzymes were also determined. The trial was conducted from June to December 2013 in a West African experimental hut design at Kolokopé, Togo.

## Methods

### Study site

This evaluation was conducted in experimental huts located at Kolokopé, Togo (07°47’59’N, 01°18’00E) from June to December 2013. The village is situated in the plateau region of the country and at 200 km from Lomé. The area is a cotton cultivation site covering approximately 236 hectares and produces an estimated 1000 tons of cotton per year. To protect farms, pyrethroid based insecticides are commonly used to spray fields [9]. The region is characterised by a long rainy season from March to October and a dry season from November to February. The annual rainfall is estimated to 1300-1500mm per year.

### WHO susceptibility Test

To characterise the wild *An*. *gambiae s*.*l*. mosquito population in Kolokopé, WHO susceptibility tests were conducted according to WHO standard protocols [10]. Mosquitoes were assayed using WHO discriminating dosages of nine insecticides belonging to four chemical classes: (1) pyrethroids (0.05% deltamethrin, 0.75% permethrin and 0.05% lambdacyhalothrin), (2) organochlorine (4% DDT), (3) organophosphates (1% fenitrothion; 5% malathion and 0.4% chlorpyrifos methyl), and (4) carbamates (0.1% propoxur and 0.1% bendiocarb). In addition, synergist assays with 5% PBO impregnated papers were conducted to determine the presence of metabolic mechanisms such as P450 enzymes. *Anopheles gambiae s*.*l*. mosquito larvae were collected in the surroundings of the village and reared to adults at the field site laboratory. Twenty to twenty-five non-blood fed female *An*. *gambiae s*.*l*., aged 3–5 days were exposed for one hour to the different insecticides and two hours specifically for fenitrothion. For the synergist assay, mosquitoes were pre-exposed to PBO for one hour before exposure to the insecticide for an additional hour. The number of mosquitoes knocked down was recorded at 60 minutes and mortality recorded after 24 hours [10]. Tests with silicone and olive oil impregnated papers were run in parallel and served as controls. Following the susceptibility tests, all mosquitoes (including controls) were kept at −20°C for further identification of *An*. *gambiae* species complex and characterization of the *kdr* mutation.

### Species identification and kdr L1014F detection

*Anopheles* specimens were randomly selected from the susceptibility testing and analyzed using SINE-PCR for species identification [11]. The detection of *kdr* L1014F was conducted following the methods of Martinez-Torres *et al*.[12] with additional confirmation using real time-PCR following the protocol of Bass *et al*. [13].

### Experimental hut design

The experimental huts are made of concrete bricks with a corrugated iron roof, a ceiling of thick polyethylene sheeting, and a concrete base surrounded by a water-filled channel to prevent entry of ants [14]. Mosquito access is via four window slits constructed from pieces of metal, fixed at an angle to create a funnel with a 1 cm wide gap. Mosquitoes fly upward to enter through the gap and downwards to exit; this precludes or greatly limits exit though the aperture enables the majority of entering mosquitoes to be accounted for. A single verandah trap made of polyethylene sheeting and screening mesh measuring 2 m long, 1.5 m wide and 1.5 m high, projects from the back wall of each hut. Movement of mosquitoes between hut and verandah is unimpeded during the night.

#### Treatment arms

Washed and unwashed LLINs were evaluated using experimental huts for their effects on free-flying, wild mosquitoes and for their ability to deter entry, repel or drive mosquitoes out of houses (i.e. induced exophily), induce mortality, and inhibit blood-feeding. Yorkool^®^ LLIN was used as a positive control and untreated polyester net was used as a negative control.

The following treatment arms were tested using seven nets per arm for the study:

1. Untreated net
2. PermaNet^®^ 3.0 unwashed
3. PermaNet^®^ 3.0 washed 20 times
4. Olyset^®^ Plus unwashed
5. Olyset^®^ Plus washed 20 times
6. Yorkool^®^ unwashed
7. Yorkool^®^ washed 20 times

#### Washing of the nets

The nets were washed according to standard WHO Phase II washing procedure. Nets were washed in aluminium bowls containing 10 litres of clean water and containing 2g/litre of soap ("Savon de Marseille") using manual agitation. Nets were rinsed twice and dried horizontally in the shade then stored at ambient temperature between daily washes. One day regeneration time was considered between each washing of all the nets following previous study results of the same treatment arms [6, 15].

Before testing in the experimental huts, the nets (including control) were deliberately holed. Six holes were made in each net: two holes in each of the long sides and one hole at each short side. Each hole measured 4cm x 4cm.

Each week, the treatment arms were rotated among the huts according to a Latin square scheme. Seven nets were used per treatment arm and each of the seven nets was tested one night during the week. At the end of the week, the huts were carefully cleaned and aired to remove potential contamination. The treatment was then rotated to a different hut.

#### Study design

Adult volunteers slept under each individual net per night. They were recruited among the inhabitants of the villages close to the site. Nets were evaluated from 23 June to 22 August 2013 for the first Latin square and from 20 October to 19 December 2013 for a second Latin square, corresponding to 98-night collections per hut to obtain sufficient number of mosquitoes for adequate statistical analysis.

Sleepers were rotated randomly among huts each night of the study. They entered a hut at dusk and remain inside until dawn. In the morning, dead and alive mosquitoes were collected from the floor of the hut as well as from the veranda traps and inside the nets; resting mosquitoes were collected using aspirators from inside the net, from the walls and roof of the hut, and veranda traps. Mosquitoes were scored by location as dead or alive and as fed or unfed. Alive mosquitoes were placed in disposable cups and provided with access to 10% sugar solution for 24 hours to assess delayed mortality.

The primary outcomes measured in experimental huts were:

- deterrence (reduction in hut entry relative to the control hut fitted with untreated nets);
- induced exophily (the proportion of mosquitoes that exited early and were found in exit traps);
- blood-feeding inhibition (the reduction in blood feeding compared with that in the control hut);
- Immediate and delayed mortality (the proportion of mosquitoes that were killed).

Outcome measures were calculated as per standard procedures. The primary analysis was a test of the non-inferiority of the candidate LLINs with PBO (PermaNet^®^ 3.0 and Olyset^®^ Plus) washed 20 times relative to the standard LLIN (Yorkool^®^) washed 20 times. According to WHOPES, a candidate LLIN is considered to meet the Phase II efficacy criteria if, after 20 washes, it performs as well as or better than the reference LN when washed 20 times in terms of blood feeding inhibition and mortality.

The percentage personal protection was calculated as follows [16]:

$$\%\;personal\;protection\;=\;100\times \frac{BFC-BFT}{BFC}$$

BFC = total number of blood fed females in the control hut

BFT = total number of blood-fed female mosquitoes in the treated hut

The insecticidal effect or overall killing effect of a treatment was calculated using the following formula [16]:

$$Overall\;insecticidal\;effect\;(\%)\;=\;100\times \frac{DT-DC}{TC}$$

DT = total number of dead mosquitoes in the treated hut

DC = total number of dead mosquitoes in the control hut

TC = total number of mosquitoes collected in the control hut

#### WHO cone bioassays

Cone bioassays were conducted according to the WHO procedures [10] on one net of each treatment arm before the first wash on the 27 May 2013, for a 2^nd^ time when all washings were completed on 17 June 2013, and for a 3^rd^ time at the end of the field experiment on the nets used in huts. For each net, 5 cones each were placed on the 5 sections of the net (roof and 4 sides). Ten females of *An*. *gambiae* s.s. Kisumu, the susceptible reference strain were introduced per cone and exposed for 3 min to the net giving an average of 50 mosquitoes. Knockdown was recorded 60 minutes after exposure and mortality was checked 24 hours after exposure. Bioassays were also conducted against wild *An*. *gambiae s*.*l*. from Kolokopé. Mosquitoes were collected at larval stage from the site, brought to the insectary, and reared until adults. WHO cone bioassays were conducted on non-blood fed adults 3 to 4 days post emergence.

#### Chemical content analysis of nets

Chemical analysis was conducted on LLIN samples pre-washing, post-washing, and post hut trial. Each net sample (10cm x 10cm) was homogenized and an analytical portion of 300mg was taken for determination of permethrin, deltamethrin, and/or PBO. Following CIPAC (Collaborative International Pesticide Analytical Council) methods deltamethrin, deltamethrin R-isomer, and PBO were extracted by heating under reflux for 60 min with xylene and were determined by gas chromatography with flame ionization detection (GC-FID) using the internal standard calibration. Permethrin and PBO was extracted in a water bath with heptane for 45 minutes and similarly determines by GC-FID.

### Statistical analysis

The analysis of each mosquito species that entered the huts was compared among the different treatment arms by a non-parametric Kruskal-Wallis test. The proportion of mosquitoes that exited early, the proportion that were killed within the hut and the proportion that successfully blood fed was compared by species and then analyzed using a logistic regression or generalized linear mixed models, which provide a framework for regression modeling of non-normal outcome data using XLSTAT software (version 2011).

### Ethics statement

In addition to approval from the traditional head of district, ethics approval was obtained from national ethics committee and the Ministry of Health of Togo. Sleeper volunteers were informed of the objective of this study and informed consent was obtained from each volunteer. A medical doctor was on hand during the trial to respond to any side effects of the treated nets or to treat any cases of fever. Any confirmed case of *P*. *falciparum* parasitaemia was treated with Coartem (artemether 20mg/lumefantrine 120 mg). Perceived adverse or beneficial side effects of the washed and unwashed nets were also noted by the seven volunteers during the experiment.

## Results

### WHO susceptibility tests

The results of the susceptibility testing in Table 1 showed that *An*. *gambiae s*.*l*. population of Kolokopé is resistant to both pyrethroids and DDT, but susceptible to organophosphates and carbamates. Among the pyrethroids, particularly low mortality (1.2%) was recorded for lambdacyhalothrin. Resistance to deltamethrin and permethrin were 14.8% and 7.5% mortalitiy respectively.

**Table 1:** Knockdown (60 min) and mortality (24 hours) of wild adult *An*. *gambiae s*.*l*. mosquitoes of Kolokopé using WHO tube test.

Insecticides	N	KD60min (%)	Mortality 24hrs (%)	Resistance status
DDT 4%	96	0	1.0	Resistant
Deltamethrin 0.05%	74	18.9	14.8	Resistant
PBO 5% + Deltamethrin 0.05%	87	100	100	Susceptible
Permethrin 0.75%	80	2.5	7.5	Resistant
PBO 5% + Permethrin 0.75%	83	85.5	92.8	Suspected resistant
Lambdacyhalothrin 0.05%	85	3.5	1.2	Resistant
Propoxur 0.1%	87	100	97.7	Suspected resistant
Bendiocarb 0.1%	85	100	98.8	Susceptible
Malathion 5%	95	100	100	Susceptible
Fenitrothion 1.0%	94	100	100	Susceptible
Chlorpyriphos methyl 0.4%	86	100	100	Susceptible

Alongside WHO susceptibility tests, synergist assays conducted with pre-exposure to PBO enhanced the mortality of permethrin from 7.5% to 92.8% and deltamethrin from 14.8% to 100%.

### Species identification and determination of resistant mechanisms

The results of the species identification and the *kdr* genotype are shown in the Table 2. Out of the 270 *An*. *gambiae s*.*l*. analyzed, 133 (49.3%) were *An*. *coluzzii* and 137 (50.7%) were *An*. *gambiae s*.*s*. The frequency of the *kdr* mutation (L1014F) was 0.62 within the population of *An*. *coluzzii* and 0.96 for *An*. *gambiae s*.*s*.

**Table 2:** Characterization of the *An*. *gambiae s*.*l*. mosquito populations from Kolokopé.

Species	N (%)	*kdr* mutation (L1014F)	N	Frequency (per species)
*An*. *coluzzi*	133 (49.3%)	RR	83	0.62
		RS	1	
		SS	49	
*An*. *gambiae s*.*s*.	137 (50.7%)	RR	131	0.96
		RS	1	
		SS	5	

### WHO cone bioassays

Full bioefficacy (meeting WHO cut-offs of >80% mortality or >95% knockdown) was observed in all unwashed and washed nets against susceptible *An*. *gambiae* Kisumu (Table 3). Bioassays against the wild resistant populations of *An*. *gambiae s*.*l*. from Kolokopé demonstrated that PermaNet^®^ 3.0 unwashed (roof portions containing deltamethrin and PBO) retained full bioefficacy before and after the hut trial (Table 4). PermaNet^®^ 3.0 washed 20 times and Olyset^®^ Plus (unwashed and washed) reported low bioefficacy against wild resistant populations. Yorkool^®^ a deltamethrin-only net delivered nearly no mortality against wild resistant *An*. *gambiae s*.*l* populations irrespective to the wash status.

**Table 3:** WHO cone bioassay against susceptible *An*. *gambiae s*.*s*. Kisumu of nets before and after washing, and after the hut trial.

Treatment arm	Unwashed		After 20 washes		After hut trial	
	KD60 (%)	Mortality (%)	KD60 (%)	Mortality (%)	KD60 (%)	Mortality (%)
1. Control	0.00^a^	0.00^a^	0.0^a^	0.0^a^	0.00^a^	0.00^a^
2. PermaNet^®^3.0 0X	100^b^	100^b^	n/a	n/a	100^b^	100^b^
3. PermaNet^®^3.0 20X	100^b^	100^b^	100^b^	100^b^	100^b^	100^b^
4. Olyset^®^ Plus 0X	100^b^	100^b^	n/a	n/a	100^b^	100^b^
5. Olyset^®^ Plus 20X	100^b^	100^b^	100.0^b^	96.3^b^	100^b^	98.03^b^
6. Yorkool^®^ 0X	100^b^	100^b^	n/a	n/a	100^b^	100^b^
7. Yorkool^®^ 20X	100^b^	100^b^	100.0^b^	94.1^b^	100^b^	96^b^

Values in the same column sharing the same letter superscript do not differ significantly (*P*> 0.05)

n/a = not applicable

**Table 4:** WHO cone bioassay against wild resistant *An*. *gambiae s*.*l*. Kolokopé of all treatment arms before and after the hut trial.

Treatment arm	Before hut trial		After hut trial	
	KD60 (%)	Mortality (%)	KD60 (%)	Mortality (%)
1. Control	0.0^a^	0.0^a^	0.0^a^	0.0^a^
2. PermaNet^®^3.0 0X –side	100.0^b^	17.9^b^	22.5^b^	5.0^a^
2. PermaNet^®^3.0 0X –roof	100.0^b^	100.0^c^	90.0^c^	100.0^b^
3. PermaNet^®^3.0 20X –side	2.6^a^	5.1^a,b^	16.2^b^	8.1^a^
3. PermaNet^®^3.0 20X –roof	80.0^b,c^	60.0^b^	10.0^a,b^	20.0^c^
4. Olyset^®^ Plus 0X	50.0^c^	15.2^b^	16.3^b^	6.1^a^
5. Olyset^®^ Plus 20X	0.0^a^	2.0^a^	2.0^a^	2.0^a^
6. Yorkool^®^ 0X	0.0^a^	0.0^a^	0.0^a^	0.0^a^
7. Yorkool^®^ 20X	0.0^a^	0.0^a^	4.0^a^	2.0^a^

Values in the same column sharing the same letter superscript do not differ significantly (*P*> 0.05)

### Experimental hut trial

In total, 4,716 mosquitoes were collected in the experimental huts during the evaluation: 2,591 (54.9%) were *An*. *gambiae s*.*l*., 1,037 (22.0%) were *Culex* species, and 1,088 (23.1%) were other species predominantly *Mansonia*. The trial results are outlined in Table 5 for *An*. *gambiae s*.*l*.

**Table 5:** Summary of trial results obtained for free flying *An*. *gambiae s*.*l*. in experimental huts (98 nights) in Kolokopé, Togo.

	Control	PermaNet^®^ 3.0–0 wash	PermaNet^®^ 3.0–20 washes	Olyset^®^ Plus 0 wash	Olyset^®^ Plus 20 washes	Yorkool^®^ 0x	Yorkool^®^ 20x
**Total females caught**	**465**^**a**^	**310**^**b**^	**348**^**b**^	**304**^**b**^	**356**^**b**^	**389**^**a**^	**419**^**a**^
Females caught per night	4.75	3.16	3.55	3.10	3.63	3.97	4.27
Deterrence (%)	-	33.33	25.16	34.62	23.44	16.34	9.89
**Total females inside verandah**	**61**^**a**^	**174**^**d**^	**159**^**b**^	**178**^**d**^	**141**^**b**^	**111**^**c**^	**96**^**c**^
Exophily (%)	13.12	56.13	45.69	58.55	39.61	28.53	22.91
95% confidence interval	10.05–16.19	50.60–61.65	40.46–50.92	53.01–64.09	34.53–44.69	24.05–33.02	18.89–26.94
Induced exophily (%)	-	49.50	37.49	52.29	30.49	17.74	11.27
**Total females blood fed**	**391**^**a**^	**16**^**c,e**^	**26**^**c**^	**10**^**e**^	**43**^**b**^	**77**^**f**^	**137**^**d**^
Blood fed (%)	84.09	5.16	7.47	3.29	12.08	19.79	32.70
95% confidence interval	80.76–87.41	2.70–7.62	4.71–10.23	1.28–5.29	8.69–15.46	15.83–23.75	28.21–37.19
Blood feeding inhibition (%)	-	93.86	91.11	96.09	85.64	76.46	61.11
**Overall mortality**	**9**^**a**^	**257**^**e**^	**226**^**b,d**^	**215**^**d**^	**206**^**b**^	**224**^**b**^	**182**^**c**^
Overall mortality (%)	1.94	82.90	64.94	70.72	57.87	57.58	43.44
95% confidence interval	0.68–3.19	78.71–87.09	59.93–69.96	65.61–75.84	52.74–62.99	52.67–62.49	38.69–48.18
Corrected mortality (%)	-	80.52	61.53	66.56	55.05	55.24	40.67

Letters in the same row sharing a letter superscript do not differ significantly (*P*> 0.05)

### *An*. *gambiae* *s.l.*

#### Control hut

During the 98-night collections, 835 culicidae were collected in the control hut. Among them 465 *An*. *gambiae s*.*l*. were recorded. A mean number of 5 *Anopheles* females were caught per night and 84.1% of them were blood fed. This corresponded to an average of 3.9 bites per man per night in the control. Natural exophily (13.1%) and natural mortality remained low throughout the experiment (1.9%).

#### Treated huts

A significant reduction in entry rates (deterrence) was noted with both unwashed PermaNet^®^ 3.0 and Olyset^®^ Plus yielding a deterrence of 33.3% and 34.6%, respectively compared with the negative control (p<0.0001). The same trend was observed for washed PermaNet^®^ 3.0 and Olyset^®^ Plus with a deterrence of 25.2% and 23.4%, respectively (p<0.0001). Both unwashed and washed LLINs with PBO deterred more *An*. *gambiae s*.*l*. compared with unwashed and washed pyrethroid-only Yorkool^®^ LLIN. Notably, the deterrence of unwashed and washed Yorkool^®^ did not differ significantly compared to the untreated net (p = 0.181 and p = 0.143, respectively).

High exophily rates were induced by PermaNet^®^ 3.0 and Olyset^®^ Plus. In contrast, exophily rates were low for the Yorkool^®^ washed and unwashed arms (28.5% and 22.9% respectively).

A decrease of the number of blood fed mosquitoes was observed with all six treatments and especially with unwashed PermaNet^®^ 3.0 and Olyset^®^ Plus, recording 93.9% and 96.1% blood feeding inhibition, respectively. However, the bloodfeeding inhibition of both unwashed LLINs with PBO were not significantly different (p = 0.263), the blood feeding inhibition of PermaNet^®^ 3.0 washed 20 times was significantly higher (91.1%) than Olyset^®^ Plus washed 20 times (85.6%) (p = 0.043).

A significantly higher corrected mortality of 80.5% was observed with PermaNet^®^ 3.0 unwashed arm compared to other treatments (p<0.05). The corrected mortality of Olyset^®^ Plus unwashed was 66.6%. As with other parameters washed Yorkool^®^ LLIN performed the worst with a corrected mortality of 40.7%.

#### Personal protection and insecticidal effect

The personal protection rates measured for PermaNet^®^ 3.0 unwashed and washed were 95.9% and 93.4% respectively; Olyset^®^ Plus unwashed and washed, 97.4% and 89.0% respectively and Yorkool^®^ unwashed and washed, 80.3 and 65/0% respectively.

The insecticidal effect showed a similar trend: PermaNet^®^ 3.0 unwashed and washed, 55.3% and 46.7% respectively; Olyset^®^ Plus unwashed and washed, 44.3% and 42.4% respectively and Yorkool^®^ unwashed and washed, 46.2% and 37.2% respectively.

#### Other species

Similar outcomes like those of *An*. *gambiae s*.*l*. were noted for all the other species including *Culex* and *Mansonia* species for induced exophily, blood feeding inhibition and mortality parameters. Data on *Culex* species are available from S1 Table.

### Chemical analysis

The mean concentration of deltamethrin, permethin, and PBO against the target concentration indicated by the manufacturer is outlined in Table 6. At the start of the trial, prior to any washing, all LLINs reported active ingredient concentrations within the target range provided by the manufacturer. Substantial active ingredient loss was noted on the side portions (deltamethrin only) of PermaNet^®^ 3.0. A similar loss of deltamethrin was also observed with Yorkool^®^ net. Also, the loss of PBO active ingredient after 20 washes was 44.0% and 56.6% lower than the initial concertation for Olyset^®^ Plus and PermaNet^®^ 3.0, respectively.

**Table 6:** Active ingredient and synergist contents of each net sample before and after the experimental hut trial.

Net type	Chemical	Target concentration		Mean concentration			Loss of active ingredient (%)
		Mean	Range	0 wash	20 washes	20 washes after hut trial	
PermaNet^®^3.0	Deltamethrin (roof)	4g/kg	3.0–5.0	3.53	2.98	2.90	**17.8**
	PBO (roof)	25g/kg	19.75–31.25	23.4	15.3	13.1	**44**
	Deltamethrin (side)	2.8g/kg	2.1–3.5	2.57	0.83	0.96	**62.6**
Olyset^®^ Plus	Permethrin	20g/kg	17.0–23.0	19.8	14.0	15.1	**23.7**
	PBO	10g/kg	7.0–13	7.67	2.5	3.33	**56.6**
Yorkool^®^	Deltamethrin	1.8g/kg	1.35–2.25	1.79	0.59	0.67	**62.6**

## Discussion

*An*. *gambiae s*.*l*is the main malaria vector in Kolokopé. PCR testing for species identification found both *An*. *coluzzii* and *An*. *gambiae s*.*s* living in sympatry at similar proportions. WHO susceptibility testing conducted from larval collections determined resistance to pyrethroid insecticides and DDT. Higher *kdr* frequency was found in *An*. *gambiae s*.*s*. (96%) compared to *An*. *coluzzi* (62%). This data is in line with previous works done in Burkina Faso demonstrating a similar predominance of the *kdr* allele frequency of *An*. *gambiae s*.*s*. [17–19].

Synergist assays conducted using pre-exposure to PBO restored the susceptibility against permethrin and deltamethrin, thus indicating resistance mediated by elevated P450 mono-oxygenase mechanisms. Similar trends were observed in Côte d’Ivoire and Benin where synergist assays using PBO also indicated the involvement of P450*s* [20, 21]. Similarly, previous studies have reported *kdr* L1014F mutation [3] and other resistance mechanisms [22] in Togo. The DDT and pyrethroid resistance observed at the experimental hut site in Kolokopé is likely conferred by both *kdr* L1014F and metabolic mechanisms. However, microplate enzyme activity experiments should be conducted to further explore the level and role metabolic mechanisms play in insecticide resistance in Togo. The confirmation of elevated P450 based mechanisms as indicated by the synergist assays also demonstrates the increased efficacy expected from LLINs with PBO (PermaNet^®^3.0 and Olyset^®^ Plus) against natural resistant *An*. *gambiae s*.*l*. populations.

WHO cone bioassays conducted against susceptible *An*. *gambiae* s.s. Kisumu indicated that all LLINs met WHO cut-offs of greater than 80% mortality or 95% knockdown before and after the hut trial. Against resistant wild *An*. *gambiae s*.*l*. only PermaNet^®^ 3.0 (roof) unwashed and washed 20 times were able to exceed 80% mortality performance cut-off. Olyset^®^ Plus delivered overall low to minimal bioefficacy against wild pyrethroid resistant *Anopheles* populations. Washed and unwashed Yorkool^®^ nets were found to have no bioefficacy against the resistant wild *An*. *gambiae s*.*l*. population, even though full bioefficacy was noted against susceptible *An*. *gambiae s*.*s*. This suggests that pyrethroid-only Yorkool^®^ nets would not be effective in this area and would perform like an untreated net.

PermaNet^®^ 3.0 and Olyset^®^ Plus exhibited high mortality and high blood feeding inhibition in free flying population of *An*. *gambiae s*.*l*. A similar trend was observed in deterrence and exophily rates of PermaNet^®^ 3.0 and Olyset^®^ Plus compared to the pyrethroid-only Yorkool^®^. These findings are in accordance with studies of Corbel *et al*. [15], Tungu *et al*. [23] and Koudou *et al*. [24] that demonstrated that PermaNet^®^ 3.0 fulfils the WHOPES efficacy criteria of Phase II studies. An experimental hut study comparing Olyset^®^ Plus and Olyset^®^ net also reported similar evidence for the advantage of incorporating PBO with pyrethroid insecticides in a LLIN for increased efficacy [25].

Yorkool^®^ net performed markedly worse than the other LLINs tested on all parameters measured. In an area with pyrethroid resistant malaria vectors, PermaNet^®^ 3.0 and Olyset^®^ Plus (LLINs with PBO) can provide additional protection in terms of reduction in blood feeding and increase in mosquito mortality, compared to a pyrethroid-only net.

The different amount of active ingredient noted for all three LLINs after analysis are similar to some previous studies [15, 25] and as reported in WHOPES reports [6, 7]. As observed in the WHOPES report, PermaNet^®^ 3.0 deltamethrin was lost more substantially from the sides than the roof of the net. It is noted that a study published after this evaluation reported a two day regeneration time for Olyset^®^ Plus [25]. While a one day washing interval was used for this study, the chemical content analysis for both permethrin and PBO were in line with previous reports [25]. Similarly, the WHOPES report for Yorkool^®^ reported 21.6% retention following 20 washes [26]; this study reported a 62.6% loss of deltamethrin, which corresponds to a retention rate of 37.4%. The percentage of loss of active ingredient is more considerable for deltamethrin treated nets than permethrin.

Apart from demonstrating the efficacy of PermaNet^®^ 3.0 and Olyset^®^ Plus, this is one of the first experimental hut studies on two available WHOPES approved LLINs with PBO that emphasises the potential benefit of LLINs with PBO to better control resistant malaria vectors compared to a pyrethroid-only LLIN. In an area with pyrethroid resistant malaria vectors with both *kdr* target site and P450-based metabolic mechanisms, PermaNet^®^ 3.0 and Olyset^®^ Plus (LLINs with PBO) can provide additional protection in terms of reduction in blood feeding and increase in mosquito mortality, compared to a pyrethroid-only net.

## Conclusion

To conclude, the present study showed efficacy of LLINs with PBO in experimental huts. PermaNet^®^ 3.0 and Olyset^®^ Plus showed significantly better performance against pyrethroid resistant populations of *An*. *gambiae s*.*l*. than the pyrethroid-only Yorkool^®^ LN. These results are encouraging and LLINs with PBO should be taken into consideration in malaria vector control strategies.

## Supporting information

S1 Table Summary of results obtained for free flying all other species in experimental huts (98 nights) in Kolokopé, Togo. (DOCX)
