# Supplementary material for: Efficacy of two PBO long lasting insecticidal nets against natural populations of Anopheles gambiae s.l. in experimental huts, Kolokopé, Togo
Source: PLoS One. 2018 Jul 11;13(7):e0192492. doi: 10.1371/journal.pone.0192492 (PMC6040683; doi:10.1371/journal.pone.0192492)
Supplement: S1 Table — (DOCX) [file pone.0192492.s001.docx]

**Additional File 1**

**Table 1**: Summary of results obtained for free flying all other species in experimental huts (98 nights) in Kolokopé, Togo.

|  | **Control** | **PermaNet® 3.0 0x** | **PermaNet® 3.0 20x** | **Olyset® Plus 0x** | **Olyset® Plus 20x** | **Yorkool® 0x** | **Yorkool® 20x** |
| --- | --- | --- | --- | --- | --- | --- | --- |
| **Total females caught** | **370^a^** | **266^b^** | **286^b^** | **252^b^** | **300^b^** | **306^a^** | **345^a^** |
| Females caught per night | 3.77 | 2.71 | 2.92 | 2.57 | 3.06 | 3.12 | 3.52 |
| Deterrency (%) | - | 28.11 | 22.70 | 31.89 | 18.92 | 17.30 | 6.76 |
|  |  |  |  |  |  |  |  |
| **Total females inside verandah** | **45^a^** | **153^b,c^** | **124^b,d^** | **153^c^** | **152^b^** | **119^d^** | **55^a^** |
| Exophily (%) | 12.16 | 57.52 | 43.36 | 60.71 | 50.67 | 38.89 | 15.94 |
| 95% confidence interval | 8.83-15.49 | 51.58-63.46 | 37.61-49.10 | 54.68-66.74 | 45.01-56.32 | 33.43-44.35 | 12.08-19.80 |
| Induced exophily (%) | - | 51.64 | 35.51 | 55.27 | 43.84 | 30.43 | 4.30 |
|  |  |  |  |  |  |  |  |
| **Total females blood fed** | **273^a^** | **2^e^** | **38^c^** | **0^e^** | **21^b^** | **65^f^** | **141^d^** |
| Blood fed (%) | 73.78 | 0.75 | 13.29 | 0.00 | 7.00 | 21.24 | 40.87 |
| 95% confidence interval | 69.30-78.27 | -0.29-1.79 | 9.35-17.22 | 0.00-0.00 | 4.11-9.89 | 16.66-25.82 | 35.68-46.06 |
| Blood feeding inhibition (%) | - | 98.98 | 81.99 | 100.00 | 90.51 | 71.21 | 44.61 |
|  |  |  |  |  |  |  |  |
| **Overall mortality** | **6^a^** | **215^b^** | **210^c^** | **222^b^** | **250^b^** | **187^e^** | **70^d^** |
| Overall mortality (%) | 1.62 | 80.83 | 73.43 | 88.10 | 83.33 | 61.11 | 20.29 |
| 95% confidence interval | 0.33-2.91 | 76.10-85.56 | 68.31-78.55 | 84.10-92.09 | 79.12-87.55 | 55.65-66.57 | 16.05-24.53 |
| Corrected mortality (%) | - | 78.20 | 71.33 | 84.92 | 82.00 | 58.50 | 18.26 |

Letters in the same row sharing a letter superscript do not differ significantly (*P*> 0.05)
